# Supplementary material for: The accuracy of healthcare worker versus self collected (2-in-1) Oropharyngeal and Bilateral Mid-Turbinate (OPMT) swabs and saliva samples for SARS-CoV-2
Source: PLoS One. 2020 Dec 16;15(12):e0244417. doi: 10.1371/journal.pone.0244417 (PMC7744114; doi:10.1371/journal.pone.0244417)
Supplement: S1 File — (PDF) [file pone.0244417.s001.pdf]

Provisional Protocol for Saliva sample collected in Lucence SAFER kit

Reagents:

Liquefaction reagent (1M DTT in 1x PBS) :

- Stock solution 1x PBS pH 6.8
- Prepare fresh: Weigh out 1.54g of DTT power and add into a 50ml tube. Dissolve in 8ml 1 X PBS and top up to 10ml mark with 1xPBS (this is sufficient for 12 samples)
- DTT: Goldbio CAS no (27565-41-9 / 3483-12-3)

Extraction kit:

- PerkinElmer® Nucleic Acid Extraction Kit (SY609)

Real-time PCR kit:

- PerkinElmer® SARS-CoV-2 Real-time RT-PCR Assay (COVID-19-PCR-AUS-C)

Instrument:

- PerkinElmer® Pre-Nat II Automated Workstation (For Extraction and PCR setup)
- Thermo Fisher Scientific QuantStudio™ 5 Real Time PCR System

<u>Procedure</u>

1) Add 750ul of liquefaction solution into each saliva sample (containing 2ml saliva and 2ml stabilization solution)
2) Vortex samples for 20 s and let it stand at room temperature for at least 15minutes prior to processing.
3) Aliquot appropriate amount of for processing. Minimal, 700ul is aliquoted to secondary tube to be processed on Pre-Nat II following manufacturer's instructions. 400ul of samples will be processed on the Pre-Nat II
4) Sample is eluted in 60ul of elution buffer (Tris-HCL)
5) Final reaction volume: 20ul of master mix and 40ul of eluted RNA.
6) Tubes are cap and run on QuantStudio 5 following manufacturer's instructions.

Run and analysis parameters for QuantStudio™ 5

Experiment method:

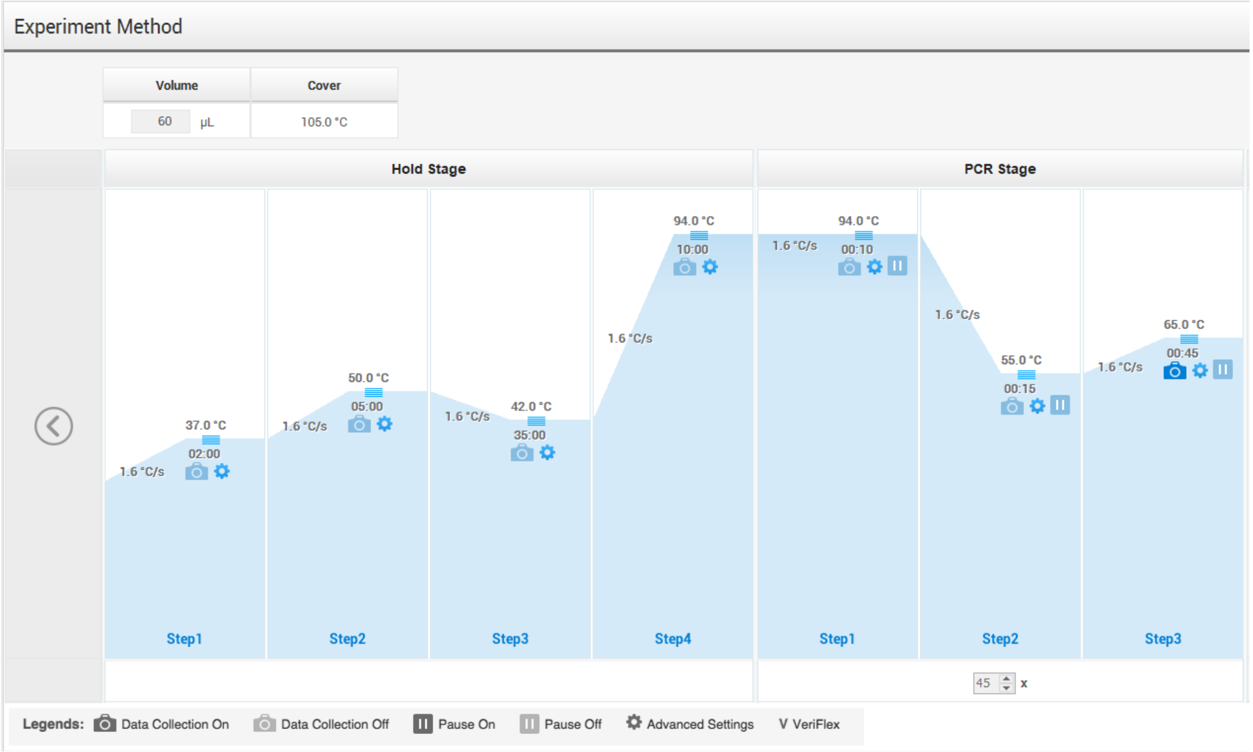

Targets assignment:

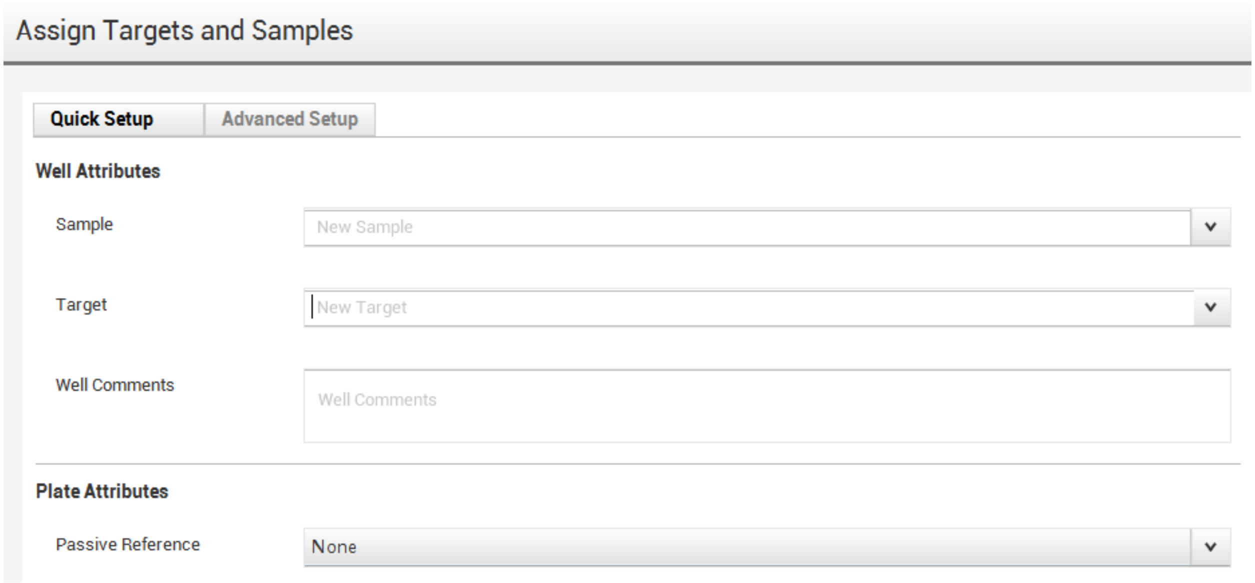

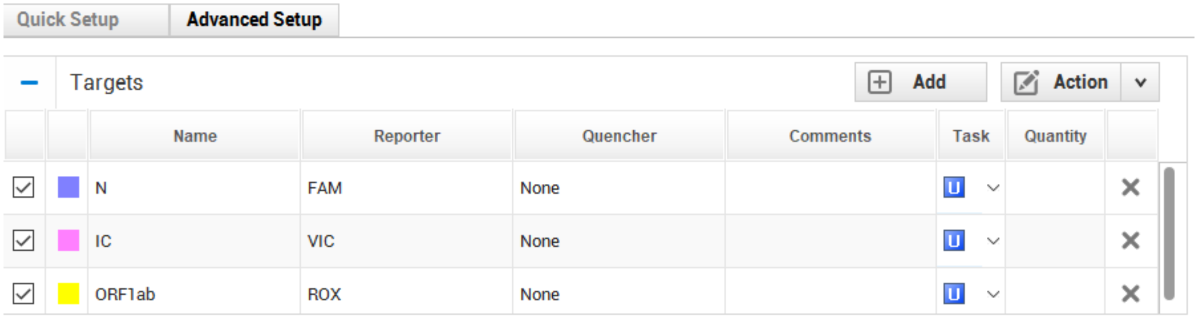

Analysis Parameters:

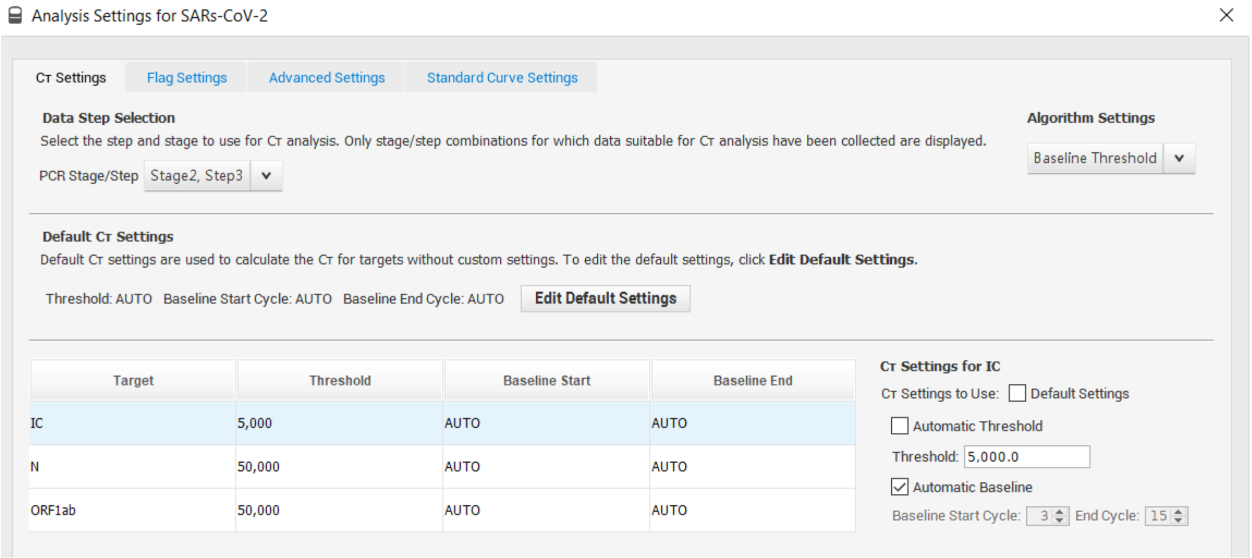

Results analysis:

User would still need to check amplification curve that they follow the sigmoidal curve for proper amplification before interpretation based on the Ct value.

Control	Ct		
	N (FAM)	ORF1ab (ROX)	IC (HEX/VIC)
Negative	Undet or > 42	Undet or > 42	Ct ≤ 40
Positive	≤ 35	≤ 35	/

Undet: Undetermined;

Ct		Result interpretation
IC (VIC/HEX)	N (FAM), ORF1ab (ROX)	
≤40	Both targets Undet or >42	SARS-CoV-2 not detected
/	Both targets ≤ 42	SARS-CoV-2 detected
/	One of the targets ≤ 42	Specimen needs to be re-tested from re-extraction
>40 or Undet	Both targets Undet or >42	Invalid result, specimen needs to be re-tested from re-extraction or re-collected from patient for test.

Undet: Undetermined;

/: No requirements on the Ct value;
