# Supplementary material for: The accuracy of healthcare worker versus self collected (2-in-1) Oropharyngeal and Bilateral Mid-Turbinate (OPMT) swabs and saliva samples for SARS-CoV-2
Source: PLoS One. 2020 Dec 16;15(12):e0244417. doi: 10.1371/journal.pone.0244417 (PMC7744114; doi:10.1371/journal.pone.0244417)
Supplement: S2 File — (PDF) [file pone.0244417.s002.pdf]

Study protocol

20/05/2020

Principal Investigator:

Dr Tan Seow Yen
Changi General Hospital
2 Simei Street 3, Singapore 529889

Site Principal Investigators:

A/Prof Toh Song Tar
Singapore General Hospital
Outram Road, Singapore 168753

Dr Ernest Lim
Woodland Health Campus
2 Yishun Central 2, Tower E, Level 5
Singapore 768024

CONFIDENTIAL

Financial Sponsors:

Sheares Healthcare Group Pte Ltd
1 Wallich Street, #32-02, Guoco Tower, Singapore 078881
Email: contact@sheareshealthcare.com.sg.

Temasek Foundation
Temasek Shophouse , 28 Orchard Road, Singapore 238832
Email: TFadmin@temasekfoundation.org.sg

PROTOCOL SIGNATURE PAGE

Protocol Title: Clinical Research Evaluating the Accuracy of Self-Collected Samples for SARS-CoV-2 compared to Healthcare Worker-Collected Samples

Protocol Number: NA

Protocol Version/ Date: 3.0 dated 20/05/2020

Sponsor Name: Sheares Healthcare Group Pte Ltd & Temasek Foundation

Declaration of Investigator

I confirm that I have read the above-mentioned protocol and its attachments. I agree to conduct the described study in compliance with all stipulations of the protocol, regulations and ICH E6 Guideline for Good Clinical Practice (GCP).

CONFIDENTIAL

Principal Investigator Name: ____

Principal Investigator Signature: ____

Date: ____

STUDY SUMMARY

Title	Clinical research evaluating the accuracy of self-collected samples for SARS-CoV-2 compared to healthcare worker-collected samples
Methodology	Cross-sectional study
Study duration	Estimated duration is approximately 3 weeks
Study centres	Changi General Hospital (CGH)<br>Singapore Expo Community Care Facility (SingHealth blocks)<br>Singapore Expo Community Care Facility (Woodland Campus blocks)
Objectives	Primary Objective: To evaluate the accuracy of self-collection of saliva sample and (2-in-1) oropharyngeal (OP) and mid-turbinate (MT) swab for SARS-CoV-2 yield sensitivity compared with healthcare worker-collected (2-in-1) OP and MT swab.<br><br>Secondary Objective: To evaluate the correlation of PCR Cycle Threshold (Ct) values of self-collected saliva samples and swabs with comparator healthcare worker-collected swabs.
Number of subjects	400 completed subjects
Inclusion and exclusion criteria	Inclusion criteria<br>• Male and female patients, ≥ 21 years-old<br>• Tested positive for COVID-19<br>• Admitted to facility within previous 3 days<br>• Ability to provide informed consent<br>• Compliance with all aspects of study protocol, methods and provision of samples<br>• Ability to read and understand English or Bengali<br><br>Exclusion criteria<br>• Nosebleeds in past 24 hours<br>• Previous nasal surgery in past 4 weeks<br>• Acute facial trauma within 8 weeks<br>• Unable to demonstrate understanding of study and instructions<br>• Experienced severe adverse reactions on prior nose and/or throat swabs<br>• Not willing to have all 3 samples collected
Study procedure	• Treatment 1: (2-in-1) OP and MT swab done by subject (Sample 1)<br>• Treatment 2: Saliva self-collected by subject (Sample 2)<br>• Control: (2-in-1) OP and MT swab done by healthcare worker (Sample 0)
Statistical methodology	Primary Endpoint<br>Equivalence or Non-inferiority comparisons of SARS-CoV-2 PCR test positivity of various samples<br><br>Secondary Endpoint<br>Correlational analysis of sample yields as measured by PCR Ct values

CONFIDENTIAL

Purpose:

To evaluate the accuracy of self-collection of saliva sample and (2-in-1) oropharyngeal (OP) and mid-turbinate (MT) swab for SARS-CoV-2 yield sensitivity compared with healthcare worker-collected (2-in-1) OP and MT swab.

Background:

The current “gold standard” for testing for SARS-CoV-2 requires health care workers to collect a nasopharyngeal (NP) sample from a patient. NP sampling reduces clinical efficiency as it is uncomfortable for patient and requires the use of personal protective equipment that are in limited supply. In a recent study, it was shown that patient-collected nasal and mid-turbinate samples demonstrated high sensitivity for SARS-CoV-2 detection using health care worker-collected NP samples as the comparator.[1] Saliva samples registered lower sensitivity in the same study.

In a recent study (yet to be published) by the National Centre for Infectious Diseases (NCID), it was concluded that a combination of OP and MT swabs is equivalent in sensitivity to an NP swab within 7 days of presenting with Covid-19 symptoms. This study explores the equivalency of SARS-CoV-2 detection subject-collected test samples compared with OP+MT combination swabs done by healthcare workers.

Furthermore, literature as shown that the viral load of SARS-Cov-2 was highest soon after symptoms onset[2,3,4], with studies showing the viral shedding occurs 2-3 days prior to onset of symptoms[1]. This indicates that the infectiousness starts 1-7 days prior to symptoms onset and peaks around days 0-2 before symptom onset[2]. This high viral load on presentation strongly suggests the easy transmission of SARS-Cov-19 even through patients with mild or no symptoms, accounting for the infective clusters found within families, religious gatherings, works places etc[2]. Viral loads decrease monotonically towards the detection limit by day 21[2].

Analyses of the viral nucleic acid shedding pattern found in infected SARS-CoV-2 patients suggest similarities to that of the influenza virus[2]. The recent NCID study mentioned above concludes that in the pneumonic stage or later disease (defined by >8 days after onset of symptoms), upper respiratory specimens perform poorly.

The scientific community world-wide is in active search for an even less invasive means of sample collection such as saliva. In a recent study by Yale University, it was suggested that a large volume sample of saliva collected from COVID-19 inpatients can be more sensitive for SARS-CoV-2 detection than NP swabs.[5]

Should self-collection of samples be validated as a viable means of SARS-CoV-2 detection, it will greatly improve our testing capacity—an important step forward in our on-going epidermic response.

CONFIDENTIAL

[1] Tu et al. 2020 April https://doi.org/10.1101/2020.04.01.20050005
[2] He et al. 2020 April https://doi.org/10.1038/s41591-020-0869-5
[3] Zou et al. 2020 Mar 382;12 NEJM
[4] To et al. 2020 Mar https://doi.org/10.1016/S1473-3099(20)30196-1
[5] Wyllie et al. 2020 April https://doi.org/10/1101/2020.04.16.20067835

Study Goals:

1. To evaluate the accuracy of self-collection of (2-in-1) OP and MT swabs for SARS-Cov-2 yield sensitivity compared with healthcare worker-collected (2-in-1) MT and OP swabs.
2. To evaluate the accuracy of self-collected saliva sample for SARS-CoV-2 yield sensitivity compared with healthcare worker-collected (2-in-1) OP and MT swabs.
3. To evaluate the correlation of PCR Cycle Threshold (Ct) values of self-collected saliva samples and swabs with comparator healthcare worker-collected swabs.

Duration of study:

The study is estimated to complete within 3 weeks of study initiation. Enrolment will remain open until the study goal is met. Duration of study for each subject will be a maximum of 30min in 1 encounter.

As the Covid-19 situation is currently quite dynamic and the Ministry of Health's policies regarding screening and decantment of patients to different types of facilities may change from the time of writing, there is a chance that the assumed daily number of Covid-19 positive subjects conveyed to study site may be lower from what has been assumed. In this case, we may need to extend the duration of study beyond 3 weeks until target sample size has been met.

CONFIDENTIAL

Methods:

Study Design.

Cross-sectional study involving 400 subjects who have been tested positive for Covid-19. Subjects will undergo three simultaneous test sample collection procedures in the following sequence:

- Subject will self-collect a sample combining OP and bilateral MT swabs using the same swab stick
- A trained healthcare worker will collect a sample combining OP and bilateral MT swabs using the same swab stick
- Subject will self-collect a saliva sample

Synthetic fibre swabs will be used for collection of OP and MT samples by both subject and healthcare worker, while SAFER-Sample (by Lucence Diagnostics) will be used to collect saliva samples.

Samples will be processed on the same day for RT-PCR testing. Data on COVID-19 PCR results (positive/negative and Ct value) will be transmitted by excel spreadsheet from lab to investigators for analysis.

Study population and selection criteria.

All aspects of the study and consent forms will be IRB approved prior to implementation. All participants will require full informed consent, be willing and able to comply with all study requirements. People admitted to study site within the previous 3 days with positive Covid-19 test and who are at least 21 years-old will be eligible for participation in this study. Those

enrolled will be the ones who are willing and able to participate in self-swabbing of two sites (nasal turbinates and throat) and produce a saliva sample, and allow healthcare worker's collection of samples from the same swab sites. People who cannot demonstrate an understanding of the study and instructions, who are not willing to participate in the collection of all three samples, had a history of nose bleeds in the last 24 hours, had recent nasal surgery in the past 4 weeks, had acute facial trauma, or who have experienced severe adverse reactions on prior nose and/or throat swabs will be excluded from the study.

Recruitment methods.

Subjects will be identified through patients recently admitted into study site for confirmed or suspected Covid-19. Recruitment of the Covid-19 positive patients will be performed within 3 days of admission to the respective study site. Informed consent will be obtained by delegated study team member within 1 day of receiving positive Covid-19 test results or latest within 3 days of admission to study site.

Sample collection

After giving consent, subject will be shown an instructional video on self-swabbing to collect OP and MT combined sample.

CONFIDENTIAL

The combined OP and MT swab is to be collected using a synthetic fibre swab with plastic shafts (ThermoFisher Traditional Swab Kit (R12552)). OP or throat swabs are to be obtained by swabbing the posterior pharynx under direct vision, avoiding touching the tongue, teeth and gums. MT swabs are to be obtained by inserting the swab into the nostril, parallel to the palate until resistance is met at the turbinates (approximately 2 cm deep) and rotated gently several times against the nasal wall and withdrawn. This is to be repeated in the other nostrils using the same swab. The swab will be obtained in the absence of the study team and/ or healthcare workers. Study team will be available to respond to any questions that the participant might have during the process.

After self-swab samples have been collected, the combined OP and MT swab is repeated by the study team. All swabs will immediately be placed into a single sterile tube containing 3ml of viral transport media (1 tube per swab).

Next, the subject will be shown an instructional video to collect his/her saliva sample. Saliva samples are collected by asking the subject to cough from the stomach to expectorate posterior oropharyngeal saliva. This step is repeated until the amount of sample collected reaches the indicated marking on the collection vial (SAFER-Sample, Lucence Diagnostics). SAFER-Sample Stabilization fluid is poured into the vial and the solution is then mixed by gentle inversion of the container five times after screwing the cap tightly on the vial.

All samples will be triple bagged and store at room temperature in a chiller bag and transported to assigned laboratory on the same day for RT-PCR testing.

Extraction process is carried out using the PerkinElmer Nucleic Acid Extraction Kits (KN0212) and run on the Quantstudio 5 Real Time PCR system using the PreNat II Automated RNA Extraction Kits. Extraction of swab samples will follow the indicated protocol for oropharyngeal swabs, while extraction of saliva samples will follow provisional protocol given to the appointed lab by manufacturer.

Study team members involved in the conduct of study procedures (i.e. sample collection etc) will undergo infection control training by qualified personnel at the study site. Study team members will be provided with the appropriate personal protection equipment to carry out the procedures.

Expected outcomes.

It is our expectation that given proper instruction, self-swabbing of throat and nasal turbinates will be found to be an accurate alternative to the healthcare worker obtained swabs of the same sites. The relative accuracy of saliva samples are exploratory at this stage. Correlation of Ct values of various means of collecting test samples are expected to be high. Data interpretation and statistical significance of results are described below.

Adverse reactions.

Most common adverse reaction is minor epistaxis. Some discomfort and gagging may be experienced

All participants will be given access to medical staff and healthcare facilities at the study sites in the event of epistaxis.

<u>Reasons for withdrawal or termination</u>

CONFIDENTIAL

A subject may be terminated from the study if the Investigator feels that it is not in the subject's best interest to continue. The following is a list of possible reasons for study discontinuation:

- Subject withdrawal of consent
- Subject is not compliant with study procedures
- Adverse event that in the opinion of the investigator would be in the best interest of the subject to discontinue study participation

All subjects are free to withdraw participation at any time, for any reason, specified or unspecified, and without prejudice. Reasonable attempts will be made by the investigator to provide a reason for subject withdrawals. The reason for the subject's withdrawal from the study will be specified in the documentation.

SAMPLE SIZE JUSTIFICATION

Basing on the study's aim that self-swabbing of throat and nasal turbinates are to be as accurate to the healthcare worker obtained swabs, Table 1 shows the error of detection for an 100% equivalence.

Table 1. Equivalence: Postulate that the difference is zero or very small

Sample size	Lower 95% CI	Error %
300	98.77%	1.23%
400	99.08%	0.92%
500	99.26%	0.74%
600	99.39%	0.61%

700	99.47%	0.53%

An error rate of less than 1% is determined to be of clinical relevance, thus a sample size of at least 400 subjects is recommended.

Since the above equivalence accuracy is exploratory, Table 2 shows a Non-Inferiority postulation that there is a difference between Self-Collected Samples for SARS-CoV-2 compared to Healthcare Worker-Collected Samples is at most 10% (otherwise consider as inferior)

Table 2. Non-Inferiority

difference	Non-inferiority region upper 95% CI <= 10%<br>Sample size
5%	>= 300
6%	>= 300
7%	>= 400
8%	>= 800

With the recommended sample size of 400 subjects, a Non-Inferiority can be achieved with at most a 7% difference for self-swabbing of throat and nasal turbinates to be compared to the healthcare worker obtained swabs,

CONFIDENTIAL

Recommendation

A sample size of at least 400 is recommended based on the above justification (see Tables 1 & 2).

STATISTICAL ANALYSIS PLAN

All analyses will be performed using SPSS 25.0 with statistical significance set at $p < 0.05$.

The estimates for the positivity results of the 3 methods will be presented as n (%).

Primary Endpoint

The 95% CI of the difference between Self-Collection and the Healthcare Worker to assess for non-inferiority (Upper 95% CI are within 10%). The differences in the accuracy of the Self-Collected Samples for SARS-CoV-2 compared to the Healthcare Worker-Collected Samples will be assessed using McNemar test. ROC analysis will be performed to assess the sensitivity, specificity, Positive Predictive and Negative Predictive of the Self-Collection swabs compared to the Healthcare Worker swabs

Secondary Endpoint

Pearson or Spearman's correlation will be presented for the association of the PCR Ct values across the 3 groups.

Data monitoring

The Investigator is responsible for verifying that data entries are accurate and correct by physically or electronically signing the CRF. The Sponsor or designee is responsible for the data management of this study including quality checking of the data.

Study monitors will perform ongoing source data verification to confirm that data entered into the CRF by authorized study site personnel are accurate, complete, and verifiable from source documents; that the safety and rights of subjects are being protected; and that the study is being conducted in accordance with the currently approved protocol and any other study agreements, ICH GCP, and all applicable regulatory requirements.

Safety Measurements

Definitions

Serious adverse event (SAE) in relation to human biomedical research, means any untoward medical occurrence as a result of any human biomedical research which:

- results in or contributes to death
- is life-threatening
- requires in-patient hospitalisation or prolongation of existing hospitalisation
- results in or contributes to persistent or significant disability/incapacity or
- results in or contributes to a congenital anomaly/birth defect
- results in such other events as may be prescribed

CONFIDENTIAL

Adverse event (AE) in relation to human biomedical research means any untoward medical occurrence as a result of any human biomedical research which is NOT serious. Adverse event can be any unfavourable and unintended sign (including an abnormal laboratory finding), symptom, or disease possibly/ probably/ definitely associated with the participant in the human biomedical research.

Collecting, Recording and Reporting of Serious Adverse Events (SAEs) to CIRB

Only related SAEs (definitely/ probably/ possibly) will be reported to CIRB. Related means there is a reasonable possibility that the event may have been caused by participation in the research. Please refer to the CIRB website for more information on Reporting Requirement and Timeline for Serious Adverse Events.

The investigator is responsible for informing CIRB after first knowledge that the case qualifies for reporting. Follow-up information will be actively sought and submitted as it becomes available.

Related AEs will not be reported to CIRB. However, the investigator is responsible to keep record of such AEs cases at the Study Site File.

Data Handling and Record Keeping

The collection of personal patient information will be limited to the amount necessary to achieve the aims of the research, so that no unnecessary sensitive information is being collected.

Only study personnel will collect data. Hard copy documents will be retained for the duration of the study until data entry.

The investigator(s)/institution(s) will permit study-related monitoring, audits and/or IRB review and regulatory inspection(s), providing direct access to source data/document.

Records and documents, including signed ICFs, pertaining to the conduct of this study must be retained by the Investigator for 7 years after study completion unless local regulations or institutional policies require a longer retention period. No records may be destroyed during the retention period without the written approval of the Sponsor. No records may be transferred to another location or party without written notification to the Sponsor.

ETHICAL CONSIDERATIONS

This study will be conducted in accordance with the ethical principles that have their origin in the Declaration of Helsinki and that are consistent with the Good Clinical Practice and the applicable regulatory requirements.

This final Study Protocol, including the final version of the Participant Information and Consent Form and recruitment materials, must be approved in writing by the Centralised Institutional Review Board (CIRB), prior to enrolment of any participants into the study.

The principle investigator is responsible for informing the CIRB of any amendments to the protocol or other study-related documents, as per local requirement.

CONFIDENTIAL

CONSENT PROCESS

The process of obtaining informed consent will be conducted in compliance with the principals of good clinical practice and requirements of the approving research ethics committee and other regulatory requirement as appropriate.

Delegated study team will approach subject with a video recording of the study which contains a simplified version of the study aim, methodology, benefit and risk. They will then be provided with hardcopy informed consent form to read. If subject is agreeable to participate in the study, the hardcopy informed consent form will be signed with the copy provided to the subject for retention. Photographs of the signed informed consent form will be taken and printed out for filing in the investigator site file. The printed informed consent form will be certified as true copy.

.

CONFLICTS OF INTEREST

Study team declares no conflicts of interest.
