# Supplementary material for: The accuracy of healthcare worker versus self collected (2-in-1) Oropharyngeal and Bilateral Mid-Turbinate (OPMT) swabs and saliva samples for SARS-CoV-2
Source: PLoS One. 2020 Dec 16;15(12):e0244417. doi: 10.1371/journal.pone.0244417 (PMC7744114; doi:10.1371/journal.pone.0244417)
Supplement: Attachment — Submitted filename: Response to Reviewers.docx [file pone.0244417.s004.docx]

**Dear Editor,**

Thank you for consideration of our manuscript. We have made considerable changes and will address your comments as well as the reviewers’ comments (in bold), point by point below.

**1. Please ensure that your manuscript meets PLOS ONE's style requirements, including those for file naming. The PLOS ONE style templates can be found at**

**https://journals.plos.org/plosone/s/file?id=wjVg/PLOSOne_formatting_sample_main_body.pdf and**

**https://journals.plos.org/plosone/s/file?id=ba62/PLOSOne_formatting_sample_title_authors_affiliations.pdf**

Have looked through the pages and the files seem to be fulfilling the requirement. Hope they are acceptable.

**2. In your Methods section, please provide additional information about the participant recruitment method and the demographic details of your participants. Please ensure you have provided sufficient details to replicate the analyses such as:**

**a) the recruitment date range (month and year),**

**b) a description of any inclusion/exclusion criteria that were applied to participant recruitment,**

**c) a table of relevant demographic details,**

**d) a statement as to whether your sample can be considered representative of a larger population, and**

**e) a description of how participants were recruited.**

The requested information have been included in the revised manuscript

**3. We note that you reference supplementary materials in your manuscript but there are no supplementary files attached. Please upload your supplementary files.**

Supplementary files have been uploaded. Apologies for missing them out in the initial submission.

**4. Comment regarding affiliation of authors SAL and CXT to commercial entities.**

I am unable to find the column to enter the amended funding statement in the entire revision process, hence the amended funding statement has been indicated in the cover letter as instructed by the video guide. The amended funding statement reads:

“This study was funded by Sheares Healthcare Group Pte Ltd.

The funder provided support in the form of salaries for authors SAL and CXT, but did not have any additional role in the study design, data collection and analysis, decision to publish, or preparation of the manuscript. The specific roles of these authors are articulated in the ‘author contributions’ section.

This does not alter our adherence to PLOS ONE policies on sharing data and materials.

Besides that, author CXT is employed by Temasek Holdings, and was acting on behalf of Sheares Healthcare Group Pte Ltd for the study. Temasek Holdings did not have any additional role in the study design, data collection and analysis, decision to publish, or preparation of the manuscript. This commercial affiliation does not alter our adherence to PLOS ONE policies on sharing data and materials.”

**Reviewer #1: In this study, Tan and colleagues present a comparison of self-collected swabs/ saliva vs. healthcare worker collected swabs. This is a controversial topic and worthy of exploration. Importantly, they find that self-collected options may be less sensitive than HCW collected swabs. The study is nicely performed and presented. It is particularly nice to see sample size analysis, which adds confidence in the conclusions. However, I do have a few recommendations to the authors to extract as much as possible from their data. I hope this helps them improve their manuscript further.**

1. **Please expand the abbreviation ‘OPMT’ when it first appears in the introduction.**

- Abbreviation expanded as per recommendation

**2. Line 89: add a note on how patients in group 1 were confirmed to have COVID-19.**

- The sentence ‘Diagnosis of COVID-19 was confirmed via a positive RT-PCR from a nasopharyngeal swab’ was added.

**3. Can I confirm that the patient swabbed both mid-turbinates using a single swab stick and then put the same swab stick into their throat?**

- Correct, the same stick is used, but first by swabbing the throat, then the bilateral mid-turbinates.

**4. It is not clear what is meant by the ‘spitting’ method? Is this posterior oropharyngeal throat saliva collection? Collecting oral fluid, spitting out saliva, and collecting posterior oropharyngeal throat saliva are all likely to have different sensitivities for SARS-CoV-2 detection, so we need to define what exactly is being collected. Would actually be nice if the instructional videos could be uploaded as supplementary material.**

- We collected posterior oropharyngeal throat saliva, which we are now calling it deep throat saliva, which we hope would clarify the nature of the saliva that is collected. We are currently unable to upload the instructional video as the producer has not given the permission to upload it as a supplementary material.

**5. Was there any particular timing of saliva collection? There is a tendency for early morning saliva to have higher viral loads (Hung DL et al, Open Forum Infect Dis, 2020).**

- There was no particular timing for saliva collection. The only restriction we placed was that the subject should not have any food/drink 30 minutes prior to the saliva collection. We decided not to limit to early morning saliva, as when the test is being used on a large scale, it is likely that patients will be tested at any time of the day. Hence the study design to simulate a real life situation.

**6. Line 130 – 131: to clarify, the sample size was calculated based on a type I error rate of 1%?**

For clarity, we have changed ' An error rate of less than 1% was determined to be of clinical relevance so a sample size of at least 400 subjects was calculated' to ‘postulating a 100% accuracy , 400 subjects will be required to achieve a lower 95% Confidence interval 99.1% (which gives a less than 1% error rate)’

**7. Line 150: redundant ‘who’**

The word “who” deleted

**8. Table 2 is not particularly useful and duplicates text in line 167 - 168. Could consider replacing with a contingency table of HCW swab vs saliva and HCW swab vs self-swab. This way, we can also check the % agreement and how many samples were detected by saliva/ self-swab, but not HCW swab.**

Added table to address the comments in both Q8 and Q9

**9. Could add a McNemar test to compare sensitivities of saliva, self-swab and self-swab + saliva against the HCW-swab ‘gold standard’.**

Added table to address the comments in both Q8 and Q9

**10. Table 3, 4: why is the total number of samples 336 (table 3), 335 (table 4)? The number of samples should be 401 – 27 (no. of negative samples) = 374? Are there missing data points?**

The original Table 3 and 4 essentially captures all those that were positive of both tests. Table 3 contains the 336 subjects where both the self-swab and HCW swab were positive, and Table 4 contains the 335 that were positive on both the saliva and HCW swab.

**11. Consider showing a scatterplot of the correlation of Ct values between self-swab and HCW swab and saliva and HCW swab.**

Have added a scatterplot to show the correlation of the Ct values of the HCW swab and self-swab, and HCW swab and saliva.

**12. Could include a column scatter plot comparing the RT-PCR Ct values of HCW swab, self-swab and saliva and statistically compare (? median) Cts of self-collected sample types to the HCW swab.**

A table showing the distribution of the Ct values in the 3 tests were included as Table 7. Using Wilcoxon Signed Rank Test, the difference in CT values between self-swab and HCW swab is statistically significance, p=0.026 similarly for the saliva and HCW swab, p<0.001. The self-swab correlates better with the HCW swab.

**Reviewer #2:**

**It is a great topic to compare the performance of self collected and HCW collected samples. But, the authors did not mention what clinical samples were used as gold standard for the diagnosis of COVID-19 in the 401 subjects. If HCW OP and MT swabs by HCW were used as gold standard, like procedure 2, listed under "test procedures", why the authors need to have a 2 stage design and include those previously tested positive for COVID-19 as subject and the second group of healthy volunteers as control? Why not include all the people when they were first tested for COVID-19 and test them with the three samples types at the very beginning?**

The diagnosis of COVID-19 in this population was made based on a positive RT-PCR on a nasopharyngeal swab. During the study period in Singapore, all patients with COVID-19 are kept isolated for at least 21 days, before returning to the community. At that point in time, where the prevalence was not high in the nation, to achieve the desired sample size of 400, we would need to sample a much larger number. Hence we elected to recruit the patients that are admitted to the community care facility and in one of the public hospital. Recognizing that with this population, we would not be able to assess the specificity of the self-swab and saliva testing, hence the second group of healthy volunteers. This approach has allowed us to be assess both the sensitivity and specificity adequately, without too high a sampling burden.

**The authors used the term 'detection rates' and 'negative correctness', are they referring to sensitivity and specificity which are more professional terms?**

Duly noted on the comment. Have changed ‘detection rates’ and ‘negative correctness’ to ‘sensitivity’ and ‘specificity’ respectively.

**Reviewer #3: Tan SY and colleagues performed a cross-sectional study to investigate healthcare worker vs. self-collected OPMT swabs and saliva samples for the detection of SARS-CoV-2 among persons with a confirmed diagnosis of COVID-19 and healthy volunteers.**

**I have some concerns as follow:**

**Major comments:**

1. **Characteristics, e.g. age, the onset of symptoms, severity of the disease, of the study populations should be included to provide readers to understand the clinical setting of the study better. The different settings may associate with different sensitivity of each specimen. One study demonstrated the lower value of saliva for the diagnosis of COVID-19 in children (Chong CY, et al. Clin Infect Dis 2020; article in press). Many studies showed more testing agreement of saliva and nasopharyngeal swab at the earlier onset of the disease (Jamal AJ, et al. Clin Infect Dis 2020; article in press, Iwasaki S, et al. J Infect 2020; article in press).**

We have added a table on the demographics of the study subjects. However, the data that we have is not complete, as some data such as date of birth could not be given to the study due to regulations from the study site. The subjects generally had mild disease, with those who are symptomatic had only upper respiratory tract symptoms, without any need for supplemental oxygen. Most subjects were within 7 days of illness onset or first positive swab.

2. **The gene that was RT-PCR test should be described. Was a housekeeping gene included in the RT-PCR reaction? The presence of the housekeeping gene in the RT-PCR test could help to determine the adequacy of a specimen collection.**

The gene that was tested was the ‘N’ gene and the ‘ORF1ab’ gene. The Ct value from the ‘N’ gene was used in the statistical analysis involving Ct values. There is an internal control that is being run is each test.

**3. In the first paragraph of the discussion, the author stated: “Our findings corroborate with existing epidemiologic data which indicates that while viral RNA detection may persist in some patients, such persistent RNA detection likely represents non-viable virus and hence, such patients are noninfectious.” The author should demonstrate the results in their study that suggest the conclusion of this statement.**

Viral cultures were not done for this study. This statement was made based on the position paper from the Academy of Medicine, Singapore as well as data from a local study (References number 9 and 10) that above the Ct values of 30, SARS CoV 2 could not be cultured. Nonetheless, as out study did not involve viral cultures, we have removed this statement from the manuscript.

**Minor comments:**

1. **The laboratory processing method that resulted in a lower yield of the saliva should be discussed. In this work, saliva samples were collected using the SAFER-Sample, which a saliva solubilizing solution was added to the samples. A preprint by Griesemer SB and coworkers demonstrated lower sensitivity to detect the virus when saliva stabilizing solution was added. Some studies showed a higher sensitivity of saliva when compare to nasopharyngeal swab. In these studies, the authors did not put either saliva stabilizing solution or viral transport media in the saliva specimen (Wylle AL, et al. N Engl J Med 2020; article in press; Rao M, et al. Clin Infect Dis 2020; article in press).**

The evidence that have emerged since our study was conducted have indicated that neat samples have given a better yield, as indicated by your good self. The initial concern was that of biosafety, and SAFER Sample was one of the first saliva collection kits to be evaluated locally when the study was being planned. We have added the discussion point that the stabilising solution might have had a negative impact on the yield of saliva testing.

2. **Please update the references. The preprints were accepted for publication.**

References number 3 and 8 updated.

3. **The writing can be improved by reorganizing the content to increase the continuity of the idea and content in the manuscript.**

e

Revised as suggested

**Reviewer #4: The manuscript by Tan et al , compared the accuracy of healthcare worker versus self collected OPMT swabs and saliva samples for SARS-CoV-2. This is a comprehensive and important study during this COVID-19 pandemic.**

**I have the following minor comments:**

**- the authors may consider adding a flowchart for the workflow and sample size of the study**

The workflow of the study process was added to the manuscript. The sample size was not included in the flowchart as there was no dropout from the study per se, but there was 1 subject that could not provide a saliva sample, hence the additional recruitment of 1 subject, giving a total number of 401 COVID-19 positive subjects.

**- the authors should include supplementary file for all single Ct values of different samples matching each patients**

Added this into the supplementary material as suggested.

**Reviewer #5:**

**In the manuscript entitled “The Accuracy of Healthcare Worker versus Self Collected (2-in-1) Oropharyngeal and Bilateral Mid-Turbinate (OPMT) Swabs and Saliva Samples for SARS-CoV-2”, Seow Yen Tan et al compares the detection rate of SARS-C0V-2 by RT-PCR in self- collected samples and samples collected by HCW. In addition, they used saliva, OP swabs and both methods combined, and compared the sensitivity of each approach in 400 samples from patients diagnosed with COVID-19 and 100 negative subjects. They concluded that saliva and sel-collected samples are inferior to OP and HCW-collected ones, respectively. However, combining both self-collected samples provided a higher detection rate.**

**Some points to discuss:**

1. **How long it took to test saliva samples after collecting them? Although swab samples were preserved in transportation medium, saliva could be affected by time until testing;**

All samples (swabs and saliva) were processed within 24 hours of collection. The samples were stored at 2-8°C. Based on the recommendations provided by the manufacturers of SAFER-sample, the samples are stable for 72 hours.

2. **Collecting saliva samples as the last procedure could affect results? Self-collected swab, followed by HCW collected swabs could interfere in the quantity and quality of saliva samples;**

We determined the sequence of testing as such, thinking that doing the swabs first, will affect the saliva yield to a lesser degree, when compared to doing saliva collection first then the swabs. This is due to the theoretical possibility that by expectorating deep throat saliva, it would potentially reduce the presence of viral material.

**3. Line 167 “self-saliva” means “self-swab”?? Please, correct it;**

Typographical error corrected

**4. In several parts of text authors refer to testing in “early phase” would provide better results: how early??In addition, there is no description on details of methodology, like time to test, preservation of samples, and characteristics of patients with positive and negative results. What about the time since diagnosis or duration of symptoms? Or severity of disease? These are important information to understand how samples were selected, and how such information could help explaining the results obtained;**

The above information were added in the revised manuscript.

5. **Authors state at conclusion that self-collection would be preferable for use in low prevalence population? Why??**

We have modified the phrasing of the statement. The thought was that in the situations where asymptomatic infection is occurring, and mass surveillance is being undertaken, an enormous pool of trained swabbers is needed to carry this out. Hence self-collection methods would be useful in situations when number of trained swabbers need to be scaled up rapidly, without a corresponding jump in infectious exposure to the staff.

6. **How author explain their results are so distinct of other reports, regarding sensitivity of saliva testing? It should be addressed in discussion. In limitations of study they cite the use of a very specific population (male migrants). How could this fact impact the results?**

This could be due to the fact that the sampling process is unique that the study design was that the subjects were already known to be positive for COVID-19. Hence the way the analysis is conducted appears unique. Most other studies would be recruiting from a testing centres where the status of the subjects are unknown and the swab done by a healthcare worker is deemed to be the gold standard. Have added this into the discussion section.

The study population is certainly not generalizable to the local population. Nonetheless, the self-collection methods is meant to be as simple as possible so that the vast majority in the population is able to follow the instructions and provide a good sample.

**Minor comments: there are several typos and gramatical mistakes. A deeper English revision is warranted.**

Revision done as advised.
