# Supplementary material for: The accuracy of healthcare worker versus self collected (2-in-1) Oropharyngeal and Bilateral Mid-Turbinate (OPMT) swabs and saliva samples for SARS-CoV-2
Source: PLoS One. 2020 Dec 16;15(12):e0244417. doi: 10.1371/journal.pone.0244417 (PMC7744114; doi:10.1371/journal.pone.0244417)
Supplement: Attachment — Submitted filename: Response to Reviewers 8 Dec.docx [file pone.0244417.s005.docx]

Dear Reviewers, thank you for the kind comments and feedback. Kindly see the responses below to your comments.

Reviewer #1: Just a few minor points:

1. **Correct grammar for this sentence: “Another study on 236 ambulatory, literate, mostly adult subjects the performance of self-collected nasal and throat swabs was at least equivalent to that of health worker collected swabs…”**

This sentence is corrected to: “In another study on 236 ambulatory subjects, the performance of self-collected nasal and throat swabs is at least equivalent to that of health worker collected swabs for the detection of SARS-CoV-2 and other respiratory viruses.”

2. **Correct grammar for this sentence: “Self-collection of samples would reduce very significantly on the reliance of trained personnel to collect samples and ramp up testing capacity.”**

This sentence is corrected to “If the self-collection of samples is proven to be a reliable alternative to a HCW swab, it would reduce the reliance of trained personnel to collect samples and enable a rapid increase in testing capacity. It would also reduce greatly the biosafety risk that is posed to HCWs and help with PPE conservation efforts.”

3. **Clarify in the methods that subjects were supervised during self-sample collection. This is hinted at in the discussion.**

Added in Line 145: “Study team members were present on site to observe and supervise the self-collection process”

4. **Line 155: inappropriately italicized ‘R’ in reverse-transcription.**

Thanks for pointing this out, this has been changed.

5. **Line 163: SARS-CoV-2, not SARS-Cov-2.**

Thanks for pointing this out, this has been changed.

6. **Line 265: Please elaborate on this statement. I can understand why the study findings would not be applicable to pediatric and very elderly populations, but the study findings should be broadly applicable to other sections of Singapore’s population?**

We made this statement as the demographic profile of the COVID-19 group (all males, generally young and healthy) is not representative of the demographics of the Singapore.

I have edited it to read: “Hence the results from this study might not be applicable to the general population, as the paediatric and very elderly were not included in the study. The migrant worker population in this study, which consist of generally young and healthy males, is also not representative of the demographics of Singapore.”

Reviewer #3: My comments have been addressed.

**However, some typos are still noted, e.g., Lines 246 and 247: the legends of figures 1 and 2 describe the same thing. The manuscript might be benifit from language editing.**

My apologies for the error, thank you for pointing this out. The legend is corrected, to reflect:

Fig 1: Correlation of Ct values of HCW swab and self-swab
Fig 2: Correlation of Ct values of HCW swab and saliva

The other typographical errors have been corrected to the best of our ability.

**In the first paragraph of the discussion, the authors stated the study on patients who are already known to be COVID-19 as the strength of the study. I think this is more likely to be the limitation, as the mean duration of the first positive swab to the study day and the mean duration between illness onset to study day were quite long. It is well known that Ct value correlated with days from symptom onset. Sensitivity of the virus detection is decreasing in saliva collected from later time of illness onset.**

**Ref: Jamal AJ, et al. Clin Infect Dis. 2020;ciaa848. Williams E, et al. J Clin Micriobiol. 2020;58(8):e00776-20.**

Agree with your point that the mean duration of the first positive swab to the study day and the mean duration between illness onset to study day were quite long hence the study design itself might have led to the results that we are seeing, where the yield of the saliva is not as good as the HCW swab. Our thought was that there was a large number of subjects who were known to be COVID-19 positive in our study, hence the perception of this being a strength.

I have restructured the discussion to improve the flow of the manuscript. The section pertaining to your comments above would read:

“The strength of our study is the large number of subjects confirmed to have COVID-19. Besides that, the study also included a high proportion of asymptomatic individuals who were picked up because of Singapore’s proactive mass screening policy. The combination of self-swab and saliva sampling performed well in these asymptomatic subjects, implying that the strategy of combined self-testing, has the ability diagnose COVID-19 in asymptomatic individuals with a sensitivity equivalent to that of a swab by a HCW. The study results from the healthy volunteers indicate a low false positive rate with self-collection methods.

These findings, indicate that self-collection methods may be a useful tool for COVID-19 surveillance in the asymptomatic individuals, and in situations where testing capacity needs to be scaled up rapidly, without a need for large increase of manpower, and without increased infectious exposure to the swabbing staff. Testing strategies can be tailored based on the target population and the intended use of the various tests on its own or in combination.

The way the study findings were presented are unlike most studies involving saliva testing for COVID-19. This is probably due to the fact that our study is carried out on subjects who are already known to have COVID-19, unlike most studies which are done in testing centres where the potential subjects’ results are still unknown. This also meant that the sampling was done later in the subjects’ trajectory of illness, as they were first tested positive for COVID-19, then enrolled into the study. The later sampling possibly had a negative impact on the sensitivity of the saliva [11].”

Reviewer #4: The authors have addressed all my concerns, i have no further questions. The manuscript should be ready to published.

Reviewer #5: (No Response)
